# Risk factors independently associated with the maintenance of severe restriction of oral intake and alternative feeding method indication at hospital outcome in patients after acute ischemic stroke

**DOI:** 10.1016/j.clinsp.2023.100275

**Published:** 2023-08-10

**Authors:** Karoline Kussik de Almeida Leite, Fernanda Chiarion Sassi, Iago Navas Perissinotti, Luiz Roberto Comerlatti, Claudia Regina Furquim de Andrade

**Affiliations:** a Oral Myology Division, Hospital das Clínicas, Faculdade de Medicina da Universidade de São Paulo, São Paulo, SP, Brazil; b Department of Physiotherapy, Speech-Language and Hearing Science and Occupational Therapy, Faculdade de Medicina da Universidade de São Paulo, São Paulo, SP, Brazil; c Department of Neurology, Hospital das Clínicas, Faculdade de Medicina da Universidade de São Paulo, São Paulo, SP, Brazil

**Keywords:** Dysphagia, Deglutition disorders, Stroke, Dysarthria, Gastrostomy

## Abstract

**Background and aims:**

From a clinical point of view, post-stroke patients present difficulties in swallowing management. The purpose of this research was to identify risk factors that were independently related to the maintenance of a severe restriction of oral intake in patients affected by acute ischemic stroke.

**Methods:**

The authors conducted a prospective observational cohort study of patients with dysphagia post-acute ischemic stroke who were admitted to an Emergency Room (ER). Demographic and clinical data were collected at ER admission. Swallowing data was based on The Functional Oral Intake Scale (FOIS) and was collected at two distinct moments: initial swallowing assessment and at the patient outcome. Patients were divided into two groups according to their FOIS level assigned on the last swallowing assessment (at hospital outcome): G1 with severe restriction of oral intake and indication of feeding tube – patients with FOIS levels 1 to 4; G2 without restriction of food consistencies in oral intake – patients with FOIS levels 5 to 7.

**Results:**

One hundred and six patients were included in our study. Results of the multivariate logistic regression model for the prediction of maintenance of a severe restriction of oral intake at hospital outcome in patients post-acute ischemic stroke indicated that increasing age (p = 0.006), and dysarthria (p = 0.003) were associated with higher chances of presenting severe restriction of oral intake at hospital outcome.

**Conclusions:**

Patients with acute ischemic stroke in an Emergency Room may experience non-resolved severe dysphagia, indicating the need to prepare for the care/rehabilitation of these patients.

## Highlights

• Dysphagia has a high incidence in post-stroke patients.
• Alternative long-term feeding method indication in post-stroke patients.
• Dysartrhia associated dysphagia in post-stroke patients.

## Introduction

Stroke is one of the leading causes of death in Brazil and is characterized as an extremely disabling disease, being considered one of the biggest public health problems in the country.^1^^,^^2^ Several factors are associated with disabilities after a stroke, such as swallowing disorders.^3^

Dysphagia has a high incidence in post-stroke patients, especially in its acute phase, contributing to the increase in mortality, morbidity and hospital costs.^1^^,^^4^^,^^5^ Studies^6^, ^7^, ^8^ have already identified that dysphagia tends to be highly prevalent in patients after acute stroke up to 90%, about half of whom remain with swallowing difficulties on hospital discharge.^9^

According to the national guidelines, during the period of hospitalization, a specialized rehabilitation team should assist post-stroke patients, aiming at early dehospitalization and avoiding or minimizing possible complications.^10^

Among the main clinical complications resulting from dysphagia are malnutrition, dehydration, and pulmonary complications such as aspiration pneumonia.^11^, ^12^, ^13^, ^14^

Due to the practical need for a prognostic instrument, the safe return of oral feeding continues to be imprecise for patients with dysphagia after stroke, based mainly on the subjective experience of the speech therapist, the physician, and the assessment of broncho-aspiration risks.^15^, ^16^, ^17^, ^18^ The difficulty in making these decisions can prolong the patient's hospitalization period, thus increasing costs for the service and risks of hospital infections.^19^^,^^20^

Considering that rehabilitation programs should be included in post-stroke patients in order to minimize the level of potential disability, the investigation of risk factors that were independently related to the maintenance of a severe restriction of oral intake in patients with ischemic stroke in the ER aims to identify the population that will need outpatient follow-up for swallowing rehabilitation after hospital discharge and alternative long-term feeding method indication, such as gastrostomy.

## Materials and methods

The authors conducted a prospective observational cohort study of patients with dysphagia post-acute ischemic stroke who were admitted to an Emergency Room (ER). The study protocol was approved by the Scientific and Ethic Committee of the Institution (Comitê de Ética para Análise de Projetos de Pesquisa do HCFMUSP), under the number 3.691.262. Informed consent was waived once the study was based on medical records analysis.

### Patient population

Patients were eligible for this study if they met all of the following criteria: a) Admission to the ER from May 2019 to May 2021; b) Acute ischemic stroke confirmed by neurological medical evaluation and CT scan; c) Bedside Swallow Evaluation (BSE) and swallow treatment requested by the primary treating physician and performed by a Speech-Language Pathologist (SLP); d) Age ≥ 18 years; e) Clinical and respiratory stability; f) Score ≥13 points on the Glasgow Coma Scale; g) Absence of previous feeding complaints or changes in diet (food consistency); h) No previous use of an alternative feeding method; i) No tracheostomy and; j) No history of surgical procedures involving the head and neck.

According to the literature the risk of death may increase by up to three times for patients who develop aspiration pneumonia during hospitalization,^21^^,^^22^ which shows the early assessment of dysphagia in post-stroke patients may minimize the risk of clinical complications.^25^ For these reasons, the protocol adopted for the present study determines that stroke patients in the acute phase should be assessed within 48 hours after hospital admission. In the present study, gold standard identification of aspiration (i.e., videofluoroscopy or fiberoptic endoscopic evaluation of swallowing) was not possible due to limitations of the clinical condition, displacement, positioning, and high cost, among others.

### Measurements-clinical assessment of swallowing

The Functional Oral Intake Scale^23^ is a 7-point ordinal scale developed to document the functional level of oral intake of food and liquid in patients with a risk of dysphagia (Table 1). For this study, the patient's swallowing ability was used to assign a specific level of FOIS based on the clinical assessment of safety and/or efficiency of eating. The professional conducting this assessment had successfully passed specific training tests. The level of FOIS was determined based on the results obtained in the Dysphagia Risk Evaluation Protocol – DREP.^24^

**Table 1:** The functional oral intake scale.

**FOIS 1**	Nothing by mouth
**FOIS 2**	Tube dependent with minimal attempts of food or liquid
**FOIS 3**	Tube dependent with consisitent oral intake of food or liquid
**FOIS 4**	Total oral diet of a single consistency
**FOIS 5**	Total oral diet with multiple consistencies, but requiring special preparation or compensations
**FOIS 6**	Total oral diet with multiple consistencies without special preparation, but with specific food limitation
**FOIS 7**	Toral oral diet with no restrictions

### Swallowing management

All patients underwent specific controlled swallowing and oral-motor rehabilitation sessions. Rehabilitation sessions were conducted based on current strategies of swallowing and oral-motor therapy. Patients were seen by a trained SLP for approximately 30 minutes per session.

### Severity of stroke

The severity of the stroke was obtained using to the National Institutes of Health Stroke Scale (NIHSS)[25] and it was calculated at the time of admission to the ER by the neurologist. The professional conducting this assessment had successfully passed specific training tests. The NIHSS^25^ is a simple instrument that can be consistently applied at the bedside by physicians, nurses, or members of a multidisciplinary team. This scale is an instrument of systematic use that allows a quantitative assessment of neurological deficits related to stroke. The scale consists of 11 items, namely: level of consciousness, eye movement, visual field, presence of facial paralysis, motor function of arms and legs, ataxia, sensitivity, language, speech, and extinction (negligence).

### Clinical indicators of swallowing functionality

Other clinical data included in the study to determine possible factors associated to the maintenance of a severe restriction of oral intake were: age; sex; laterality of the ischemic stroke (right hemisphere, left hemisphere or bilateral hemispheres); impaired cerebral circulation (anterior or carotid region – involvement of the middle and anterior cerebral arteries, posterior or vertebral basilar region – involvement of the posterior cerebral artery); previous *neurological comorbidities; stroke hemorrhagic transformation*; acute frontal operculum injury; thrombolysis at admission; thrombectomy at admission; presence of dysphagia predictores(weak/ineffective cough; cough in the water test; wet voice in the water test; dysphonia; dysarthria; altered level of consciousness; altered gag reflex; facial palsy; aphasia); pre-swallowing assessment fasting; time between swallowing assessment and return to oral feeding (in days); indications of requiring an alternative feeding method after swallowing assessment; time between swallowing assessment and recommendation to remove of the alternative feeding(in days); recommendation to remove the alternative feeding at clinical outcome; use of the alternative feeding method before swallowing assessment; in-hospital gastrostomy placement; alternative feeding method associated a oral feeding indicated after swallowing assessment; FOIS level at admission; FOIS level at outcome; patient outcome (hospital discharge, suspended swallowing therapy due to worsening of the clinical condition, hospital transfer, in-hospitlar death, death until 2 months after clinical outcome).

### Data analysis

Analysis was performed using the Stata software, version 17.0, Statacorp inc. In order to show the overall results, categorical variables were presented in contingency tables comprising absolute (n) and relative (%) frequencies, and continuous variables were described using mean and standard deviation. Patients were divided into two groups according to their FOIS level assigned on the last swallowing assessment (i.e., prior to hospital outcome-hospital transfer, hospital discharge, or in-hospital death): G1 with severe restriction of oral intake and indication of feeding tube – patients with FOIS levels 1 to 4; G2 without restriction of food consistencies in oral intake – patients with FOIS levels 5 to 7.

Quantitative data were described with mean and standard deviation (SD), and the groups were compared using Student's *t*-test for normal distribution and the Mann-Whitney Test for asymmetric distributions. Qualitative data were described as total and percentage counts and groups were compared using Mann-Whitney Tests for ordinal qualitative variables and Chi-Square Test and Fisher's Exact Test for nominal quantitative variables. The significance level adopted in all analyzes was 5%. No correction was performed for multiple comparisons.

Secondarily, the possible risk factors were analyzed to identify which items were the most significant predictors of non-resolved dysphagia at hospital outcomes in the investigated population. The backward stepwise logistic regression model was used to examine the relationships between independent variables. As previously described, the dependent variable was the maintenance of severe restriction of oral intake at hospital outcome (i.e., FOIS levels 1 to 4). Any variable having a significant univariate test at p ≤ 0.1 was selected as a candidate for the multivariate analysis. During the iterative multivariate fitting, covariates were removed from the model if they were non-significant at p ≤ 0.05 and not a confounder (i.e., did not change any remaining parameter estimates by more than 20%), using the backward stepwise selection method. The variables that remained in the model were considered independent risk factors.

## Results

During the study period, 370 adult patients (> 18 years) were admitted to the Institution with stroke, of whom 264 were excluded from this study due to a lack of eligibility criteria. From the 106 eligible patients for a swallowing assessment, 72 presented severe restriction of oral intake, unresolved dysphagia with an indication of alternative feeding method at hospital outcome – patients with FOIS levels 1 to 4 (G1), and 34 patients presented no severe restriction of oral intake, improvement of dysphagia without indication of alternative feeding method at hospital outcome ‒ patients with FOIS levels 5, 6 and 7 (G2). Demographic and clinical data are presented in Table 2.

**Table 2:** Intergroup comparison for demographic and clinical data.

	G1 (n = 72)	G2 (n = 34)	Total (106)	p-value
**Age (mean ± SD)**	72.9 (12.93)	65.38(12.87)	70.49 (12.91)	**0.004**^a^
**Female sex, n (%)**	34 (45.84%)	16 (47.2%)	49(46.23%)	0.987^b^
**Previous *neurological injury,* n (%)**	43 (59.7%)	9 (26.5%)	55(51.88%)	**0.001**^b^
**Lateralization, n (%)**				0.269^c^
Left	39(54.17%)	19(55.88%)	58(54.72%)	
Rigth	30(41.67%)	15(44.12%)	45(42.45%)	
Bilateral	3(4.16%)	0	3 (2.83%)	
**Circulation, n (%)**				0.541^c^
Carotid Territory	61 (84.72%)	31(91.18%)	92(86.79%)	
Vertebral basilar territory	11(15.28%)	3 (8.82%)	14(13.20%)	
**NIHSS, n (%)**				
0 to 5	8(11.11%)	2(5.89%)	10(9.44%)	0.513^b^
6 to 13	18(25%)	14(41.17%)	32(30.18%)	0.151^a^
> 14	46(63.89%)	18(52.94%)	64(60.38%)	**0.077**^a^
**Acute opercular injury, n (%)**	32 (50%)	17 (40.5%)	49(46.22%)	0.121^b^
**Thrombectomy on admission, n (%)**	1 (1.38%)	1 (2.94%)	2 (1.88%)	0.541^c^
**Thrombolysis on admission, n (%)**	20 (27.78%)	17 (50%)	37(34.90%)	**0.025**^b^
**Hemorrhagic transformation, n (%)**	10 (13.88%)	6 (17.64%)	16(15.09%)	0.772^c^

DP, Standard Deviation; n, number of participants; %, Percentage of participants; G1, With severe restriction of oral intake; G2, Without severe restriction of oral intake; NIHSS, National Institute of Health Stroke Scale; IQR, Interquartile Amplitude.

a Significant difference (p < 0.05) in the *t*-test.

b Significant difference (p < 0.05) in Pearson's Chi-square test.

c Significant difference (p < 0.05) in Fisher's exact test.

According to the intergroup comparison, differences were observed between the groups: Patients in G1 had a higher mean age, a higher incidence of previous neurological injury, and more severe strokes assessed by the National Institute of Health Stroke Scale^25^ (NIHSS). In addition, they underwent less intravenous thrombolysis. There was no difference between gender, stroke laterality, vascular territory, acute opercular involvement, thrombectomy, or hemorrhagic transformation.

Table 3 shows the comparison of groups according to clinical speech-language pathology variables. Patients in Group 1 had higher rates of indication of an alternative feeding method after the swallowing assessment, lower rates of indication of a mixed diet (oral diet associated with an alternative method), lower FOIS scores in the swallowing assessment, and lower FOIS scores at hospital outcomes. Group 1 also had a lower rate recommendation to remove the alternative feeding tube at the outcome and a higher rate of performing gastrostomy tube during hospitalization. No patient in Group 1 received a recommendation to remove the alternative feeding method due to the maintenance of severe impairment of oral intake, while all patients in Group 2 were able to remove the alternative method at outcome once they had an evolution of oral intake.

**Table 3:** Intergroup comparison for clinical indicators of dysphagia.

	G1 (n = 72)	G2 (n = 34)	Total (106)	p-value
**Number of days from swallowing assessment to oral feeding return (mean ± SD)**	3.33 (3.61)	2.88 (4.05)	3.11 (3.81)	0.435^d^
**Use of alternative feeding tube before swallowing assessment, n (%)**	27 (37.5%)	10 (29.41%)	37 (34.90%)	0.415^b^
**Pre-swallowing assessment fasting, n (%)**	42 (58.33%)	23 (67.64%)	65 (61.32%)	0.358^b^
**Exclusive alternative feeding tube indication after swallowing assessment, n (%)**	52 (72.22%)	12 (35.29%)	64 (60.38%)	**<0.001**^b^
**Alternative feeding method associated a oral feeding indicated after swallowing assessment, n (%)**	20 (22.78%)	22 (64.71%)	42 (39.62%)	**<0.001**^b^
**Number of days from swallowing assessment to recommendation to remove the alternative feeding indication (mean±SD)**	9 (5.43)	6.29 (5.55)	6.64 (5.38)	0.751^d^
**In-hospital gastrostomy, n (%)**	17 (23.61%)	1 (2.94%)	18 (16.98%)	**0.011**^c^
**FOIS at swallowing assessment (mean ± SD)**	2 (0.95)	2.94 (0.86)	2.3 (1.02)	**<0.001**^d^
**FOIS at hospital outcome (mean ± SD)**	2.22 (1.09)	5.97 (0.87)	3.42 (2.03)	**<0.001**^d^
**Indication to remove alternative feeding method at hospital outcome, n (%)**	0	34 (100%)	34 (32.07%)	**<0.001**^c^

DP, Standard Deviation; n, number of participants; N, Numero; %, Percentage of participants; G1, With severe restriction of oral intake; G2, Without severe restriction of oral intake; NIHSS, National Institute of Health Stroke Scale; FOIS, Functional Scale score of Oral Intake.

aSignificant difference (p < 0.05) in the *t-* test.

b Significant difference (p < 0.05) in Pearson's Chi-Square test.

c Significant difference (p < 0.05) in Fisher's exact test.

d Significant difference (p < 0.05) in the Mann-Whitney exact test.

Table 4 shows the comparison between the groups for the clinical outcome variable. Patients in Group 2 were discharged significantly more frequently, had lower in-hospital mortality (none in this group), and lower mortality less than 2 months after hospital outcome. There was a trend towards a greater number of suspended swallowing therapy due to clinical worsening in Group 1.

**Table 4:** Intergroup comparison for the hospital outcome variable.

Hospital outcome	G1 (n = 72)	G2 (n = 34)	Total (106)	p-value
Hospital discharge, n (%)	42 (58.33%)	32 (94.12%)	74 (69.81%)	<0.001^a^
Suspended swallowing therapy due clinical worsening, n (%)	8 (11.11%)	0	8 (7.54%)	0.052^a^
Hospital transfer, n (%)	7 (9.72%)	2 (5.88%)	9 (8.49%)	0.715^a^
In-hospital death, n (%)	15 (20.83%)	0	15 (14.15%)	0.002^a^
Death < 2 months post-outcome, n (%)	13 (22.81%)	1 (2.94%)	14 (15.38%)	0.014^a^

n, Number of participants; %, Percentage of participants; G1, With severe restriction of oral intake; G2, Without severe restriction of oral intake.

a Significant difference (p < 0.05) in Fisher's exact test.

Table 5 compares the groups in terms of speech-therapy data. G1 patients had a higher incidence of dysphonia, altered gag reflex, weak/ineffective cough, altered vocal production after water test, coughing with water, dysarthria, and lower level of consciousness.

**Table 5:** Intergroup comparison according to the results of the initial swallowing assessment evaluation.

	G1 (n = 72)	G2 (n = 34)	Total (106)	p-value
**Dysphonia, n (%)**	48 (66.66%)	12 (35.29%)	60 (56.60%)	0.002^a^
**Altered gag reflex, n (%)**	48 (66.66%)	16 (47.05%)	64 (60.37%)	0.054^a^
**Weak/ineffective cough, n (%)**	50 (69.44%)	13 (38.23%)	63 (59.43%)	0.002^a^
**Vocal alteration in water test, n (%)**	44 (61.11%)	11 (32.35%)	55 (51.88%)	0.006^a^
**Cough in water test, n (%)**	62 (86.11%)	18 (52.94%)	80 (75.47%)	<0.001^a^
**Dysarthria, n (%)**	66 (91.66%)	23 (67.64%)	89 (83.96%)	0.002^a^
**Aphasia, n (%)**	44 (61.11%)	15 (44.11%)	59 (55.66%)	0.100^a^
**Facial palsy, n (%)**	63 (87.5%)	33 (97.05%)	96 (90.56%)	0.116^a^
**Lowered level of consciousness, n (%)**	28 (38.88%)	6 (17.64%)	34 (32.07%)	0.029^a^

n, Number of participants; %, Percentage of participants; G1, With severe restriction of oral intake; G2, Without severe restriction of oral intake.

a Significant difference (p < 0.05) in Pearson's Chi-Square test.

Tables 6 and 7 present the results of the multivariate logistic regression model for the prediction of severe restriction of oral intake at hospital outcomes in patients with acute ischemic stroke in ER. The univariate analysis identified 10 covariates initially as potential candidates for the multivariate model at the 0.1 alpha level based on the likelihood-ratio statistic: age; previous *neurological comorbidities (i.e., dementia, stroke);* thrombolysis at admission; dysphonia; altered gag reflex; weak/ineffective cough; wet voice in the water test; cough in the water test; dysarthria; and altered level of consciousness.

**Table 6:** Multivariate regression model for prediction of maintenance severe restriction of oral intake in patients with post acute ischemic stroke ‒ 1^st^ iteration (model-full).

	Odds Ratio	95% IC		p-value
		Inferior	Superior	
**Age**	1.04	1.00	1.09	**0.046**^a^
**Previous Neurological Injury**	2.31	0.78	6.87	0.131
**Thrombolysis on admission**	0.40	0.14	1.17	0.096
**Dysphonia**	1.64	0.35	7.63	0.527
**Altered gag reflex**	1.10	0.26	4.65	0.893
**Weak/ineffective cough**	0.99	0.19	5.28	0.994
**Vocal alteration after swallowing**	0.80	0.14	4.64	0.809
**Cough in water test**	3.11	0.97	10.01	0.056
**Dysarthria**	5.29	1.19	23.46	**0.028**^a^
**Lowered level of consciousness**	1.07	0.25	4.57	**0.924**

n, Number of participants; %, Percentage of participants; G1, With severe restriction of oral intake; G2, Without severe restriction of oral intake; ER, Emergency Room; CI, Confidence Interval.

a Significant iteration (p < 0.05) in the complete multivariate logistic regression model.

**Table 7:** Multivariate logistic regression model for prediction of maintenance severe restriction of oral intake in patients with post acute ischemic stroke ‒ 2^nd^ iteration (final model).

	Odds Ratio	95% CI		p-value
		Lower	Upper	
**Age**	1.05	1.02	11.09	**0.006**^a^
**Dysarthria**	6.01	1.87	19.33	**0.003**^a^

n, Number of participants; %, Percentage of participants; G1, With severe restriction of oral intake; G2, Without severe restriction of oral intake; PS, Emergency Room; CI, Confidence Interval.

a Significant iteration (p < 0.01) in the multivariate logistic regression model ‒ stepwise method for selection.

Table 6 shows the initial results of the logistic regression model and Table 7 shows the resulting model, containing only significant covariates. This analysis indicated that increasing age and the presence of dysarthria were associated with greater odds of patients with acute ischemic stroke having maintenance of severe restriction of oral intake in the hospital outcome.

## Discussion

Overall, the present results indicated that patients included in the study had a mean age of 70.4 years, previous neurological impairment and NIHSS score ≥ 14. The majority of these patients (67.9%), were discharged from the hospital with severe impairment of oral intake. A few variables were independently associated with non-resolved dysphagia at hospital outcome: age; previous *neurological comorbidities (i.e., dementia, stroke);* thrombolysis at admission; dysphonia; altered gag reflex; weak/ineffective cough; a wet voice in the water test; cough in the water test; dysarthria; and altered level of consciousness.

The literature has documented that the most evidenced predictor of dysphagia in post-stroke patients is advanced age (over 55 years). Besides, the prevalence of swallowing disorders is higher in the presence of age-related diseases such as stroke and dementia.^10^^,^^26^^,^^27^ Corroborating to these studies, the present results show that 59.7% of individuals with dysphagia had previous neurological alterations related to dementia processes or previous stroke. Changes in swallowing physiology, such as loss of muscle mass, can result in loss of muscle strength and mobility. These changes can have a negative impact on swallowing efficiency and airway protection. Age-related atrophy of the soft tissues of the pharynx and larynx may also be considered a contributing factor to swallowing changes.^28^ Considering stroke, studies have already described that increasing age is a strong predictive factor for a stronger and faster loss of muscle activities, increasing the incidence of dysphagia and mortality.^8^^,^^10^^,^^29^ The present results show that both groups were composed of elderly patients but G1 had older participants, while G1 had a mean age of 72.9 years G2 had a mean age of 65.3.

Group 1 of the present study presented a high percentage (59.7%) of individuals with previous neurological alterations related to dementia processes and previous stroke. This result is similar to studies in which participants who failed dysphagia screening had as one of the main characteristics the highest previous stroke rate.^30^^,^^31^ In this study, the presence of a previous stroke was associated as a risk factor for dysphagia, demonstrating that the extent of the lesion may be more related. A recent meta-analysis showed that strokes are strong risk factors for the development of dementia, especially when related to the presence of multiple lesions, extensive stroke, and location in the left hemisphere.^32^ The literature also suggests that the existence of a previous dementia may predispose to stroke, due to neural inflammation and impairment of the integrity of the arterial walls, increasing the risk of cerebrovascular events and extensive stroke.^32^^,^^33^ The present results are similar to a previous study in which dementia was directly associated with more severe dysphagia.^10^ In these cases, the main alterations of swallowing are the oral phase (difficulty chewing and preparation of the food bolus, increased oral transit time) and the pharyngeal phase (delay in swallowing reflex, reduced laryngeal elevation, and fluid aspiration).^34^ This demonstrates the importance of patients with a history of dementia being prioritized for swallowing assessment and rehabilitation after stroke episodes.^10^

The results of this work showed patients with poor swallowing functionality had a worse outcome if compared with the non-dysphagia group. The present data collection was done in a tertiary hospital where clinical stability is usually not related to full dysphagia rehabilitation. Then, the patient may have hospital discharge with a good clinical health conditions but remain with an indication of speech therapy intervention.^29^ The literature shows that half of post-stroke patients still have swallowing disorders after hospital discharge. This fact can consider severe dysphagia as a predictor of higher mortality rates and unfavorable outcomes.^10^^,^^29^

Considering dysphagia indicators, patients that remained with severe restriction of oral intake at hospital outcome (G1) presented higher rates of indication of food alternative method after speech therapy evaluation, lower rates of indication of a mixed diet and lower FOIS scores. Group 1 also presented a lower rate of recommendation to remove alternative feeding tubes at hospital outcomes and a higher rate of gastrostomy indication during hospitalization. No patient of Group 1 was indicated to remove the alternative feeding method due to the maintenance of severe impairment of oral intake, while all patients in Group 2 were able to remove the alternative tube at hospital outcome once they had an evolution of oral ingestion. According to the literature, the need for an alternative feeding method in post-stroke patients also showed a correlation with a higher mortality rate and a worse outcome.^5^^,^^35^

In the present study, patients with severe restriction of oral intake presented worse scores on the NIHSS scale. The scale does not have items that directly assess swallowing, but it has parameters that help in the patient's functional clinical evaluation, such as level of consciousness, facial palsy, aphasia, dysarthria, motor, and sensory deficits. Thus, despite being a non-valid measure for the screening of dysphagia,^36^ NIHSS has been used in many studies as a complement to the swallowing assessment, demonstrating a good association with the presence of dysphagia and pneumonia.^26^ According to the literature^24^^,^^37^ a complete evaluation of dysphagia should include tests that assess changes in oral functions, such as changes in vocal quality, dysarthria, alteration in gag reflex, weak voluntary cough, and voice changes in water test. These tests should be brief, noninvasive, present low risk to the patient and identify the symptoms of dysphagia.^10^^,^^38^ The preliminary swallowing evaluation^38^ in the present institution evaluates all these aspects, and in thesample, it was observed that G1 presented a higher prevalence of patients with dysarthria, altered nauseous reflexes, and lowered level of consciousness.

Dysarthria affects 41% of the stroke population stroke^39^ and is characterized by a speech disorder due to changes in muscle control of the mechanisms involved in its production, due to facial paralysis, weakness and/or incoordination of speech muscles. Motor speech production involves muscles also recruited in swallowing physiology (lips, tongue and cheeks).^34^ Thus, the change in tone, mobility and sensitivity of this musculature seems to be directly related to dysphagia and dysarthria which shows the importance of an integrated approach to breathing, swallowing and phonation mechanisms in the speech therapy clinic.

It is important to highlight that both the presence of advanced age and the presence of dysarthria were independently associated with the maintenance of severe restriction of oral intake in patients with acute stroke and severe dysphagia evaluated in the ER. This data is in line with the findings reported in the literature,^2^^,^^10^^,^^11^^,^^17^^,^^35^ which indicate the age factor is one of the predictors of dysphagia in post-stroke patients. A previous study conducted at this same institution,^10^ with the same population, identified that patients aged ≥ 70 years had a higher prevalence of dysphagia. In thecurrent sample, only patients with severe restriction of oral intake identified in the swallowing assessment were included, and age ≥ 72 years was considered an independent risk factor for non-resolution of dysphagia. The prevalence of dysarthria was 91.66%, above the average found in the literature.^37^^,^^39^ Given the relevance of this finding, it is suggested that more studies be conducted to better explore the relationship between dysarthria and dysphagia in patients with acute ischemic stroke and in other populations.

## Conclusion

The presentresults emphasize the importance of identifying the profile of patients who remain with severe dysphagia after hospital discharge and an alternative long-term feeding method indication, such as gastrostomy. Thus, demanding outpatient follow-up for swallowing rehabilitation, aims to reduce the risk of readmissions due to clinical complications such as dehydration, malnutrition, and aspiration pneumonia.

In conclusion, the results suggest that patients with severe restriction of oral intake after acute stroke who present dysarthria and age ≥ 70, should be prioritized for early gastrostomy indication.

## Authors' contributions

Leite KKA was responsible for the data collection, analysis and interpretation of the results, and manuscript writing. Sassi FC was responsible for organizing and conducting the design of methodology, interpretation of results and writing. Perissinotti IA was responsible for the data analyses. Comerlatti was responsible for specifically critical review. Andrade CR was responsible for the research and experimental design, and contributed to manuscript preparation and revision.

## Conflicts of interest

The authors declare no conflicts of interest.
